# Supplementary material for: The effects of functional diversity on biomass production, variability, and resilience of ecosystem functions in a tritrophic system
Source: Sci Rep. 2019 May 17;9:7541. doi: 10.1038/s41598-019-43974-1 (PMC6525189; doi:10.1038/s41598-019-43974-1)
Supplement: Supplementary file 1 — Appendices [file 41598_2019_43974_MOESM1_ESM.pdf]

Supplementary information for *The effects of functional diversity on biomass production, variability, and resilience of ecosystem functions in a tritrophic system*

Ruben Ceulemans, Ursula Gaedke, Toni Klauschies and Christian Guill
University of Potsdam, Germany

A Exploration of parameter space and food web structure

The model described in the main text contains many parameters of different types. The effect of parameters which control the environmental conditions, such as the inflow nutrient concentration N_0 and the dilution rate δ will be studied in section A.1.

Other parameters relating to the species properties also influence the structure of the food web. In particular, the mass ratio $\frac{m_I}{m_B} = \frac{m_T}{m_I}$ and the allometric scaling exponent λ influence the species' maximum growth rate and thus the intensity of trophic interactions. In the main text, the mass ratio between the species of adjacent trophic levels was set to 1000, with $\lambda = -0.15$ in order to represent aquatic communities. In this case, going up in trophic level rescales the maximal growth rate by a factor of $1000^{-0.15} \approx 0.35$. It should be noted that in terrestrial communities, where mass ratios of 100 and scaling exponents of $\lambda = -0.25$ might be more representative (Brose et al., 2006), the species' maximal growth rate is scaled with a factor that is almost equal to the one for aquatic systems: $100^{-0.25} \approx 0.32$. However, in order to verify our results for different food web structures, we have looked at variations of these mass ratios while keeping the scaling exponent fixed at $\lambda = -0.15$, such that the maximal grazing rates are altered significantly (section A.2). In addition, we have also investigated what happens when we also scale the dilution rate δ allometrically, transforming it to a more traditional death rate, such that higher trophic levels experience a lower death rate.

A.1 Varying environment parameters

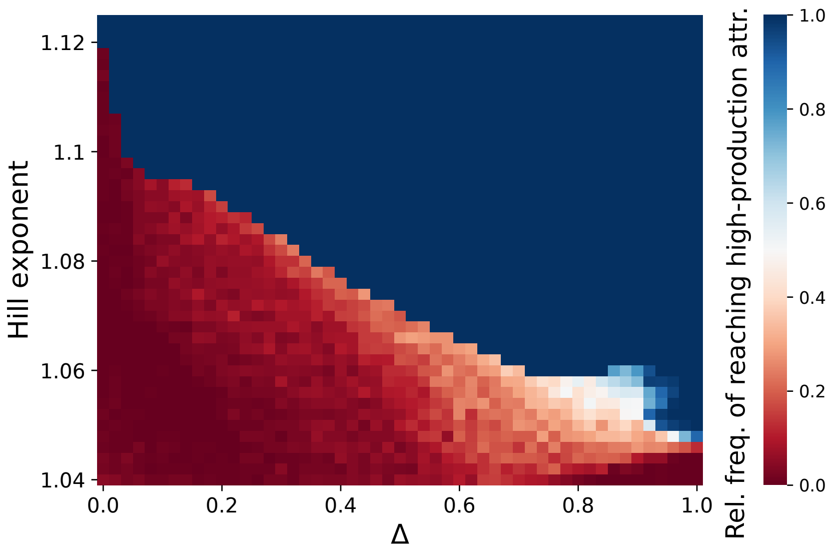

Figure A1: *Equivalent of Figure 5 (main text), when the interaction strength between the defended prey species and the selective consumer species is set to* 10^{-6} *instead of* 10^{-4}. *Comparison between these two figures shows no significant differences, emphasizing that the value of* 10^{-4} *as used in the main text is sufficiently low, and lowering it further does not alter our results.*

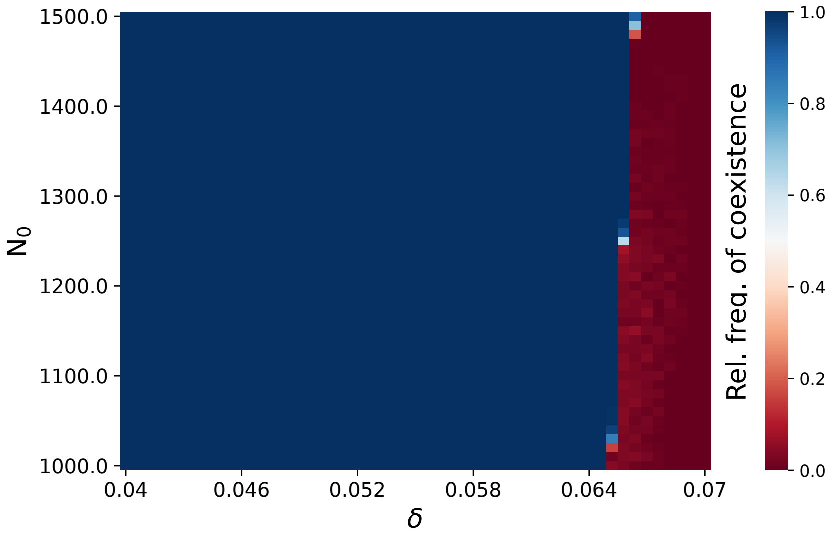

Figure A2: *Relative frequency of initial conditions leading to a state for which all species coexist for* $\Delta = 1$ *(maximal trait variation). For each combination of the inflow concentration* N_0 *and the dilution rate* δ, 200 *randomly sampled initial conditions were tested. As long as the dilution rate is small enough such that the slowest growing top species* T_n *can remain in the system, coexistence is very probable in our model, independent of these external environmental factors.*

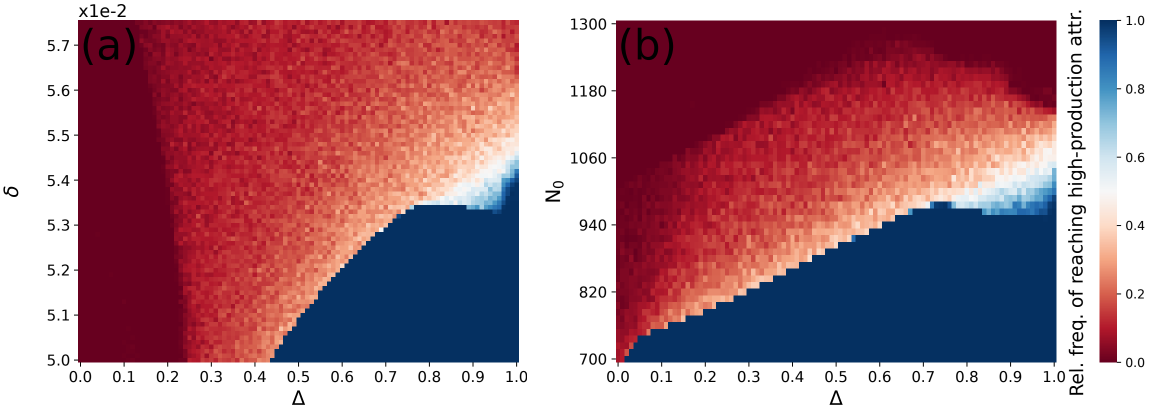

Figure A3: *(a) Relative frequencies of reaching the high-production attractor, as a function of the trait difference* Δ *and the dilution rate* δ*, for* $h = 1.05$*. Each of the points in the* 101×76 *grid shows the relative frequency of reaching the high-production attractor, sampling* 200 *random initial conditions. The patterns described in the main text are also present as* δ *is varied. The basin of attraction generally increases in size as the trait difference increases, meaning the high-production state is more resilient against pulse perturbations. In addition, the high-production attractor's boundary crisis is present over the whole range of* δ *when trait differences are low, hence, higher trait differences also protect the high-production state against press perturbations. However, as* δ *increases the high-production attractor becomes less resilient, possibly due to the high sensitivity of the top species to the dilution rate, as they have the lowest growth rates. (b) Relative frequencies of reaching the high-production attractor as a function of the trait difference* Δ *and the nutrient inflow concentration* N_0*, for* $h = 1.05$*. Each of the points in the* 101×51 *grid shows the relative frequency of reaching the high-production attractor. The basin of attraction generally increases in size as the trait difference increases, meaning the high-production state is more resilient against pulse perturbations. However, for increasing nutrient inflow concentration, there is a clear trend towards the more variable low-production state, and eventually the high-production state disappears through a boundary crisis as* N_0 *is increased further. This result is in accordance with the paradox of enrichment, a commonly observed effect where a consumer-resource system is destabilized when food becomes more available.*

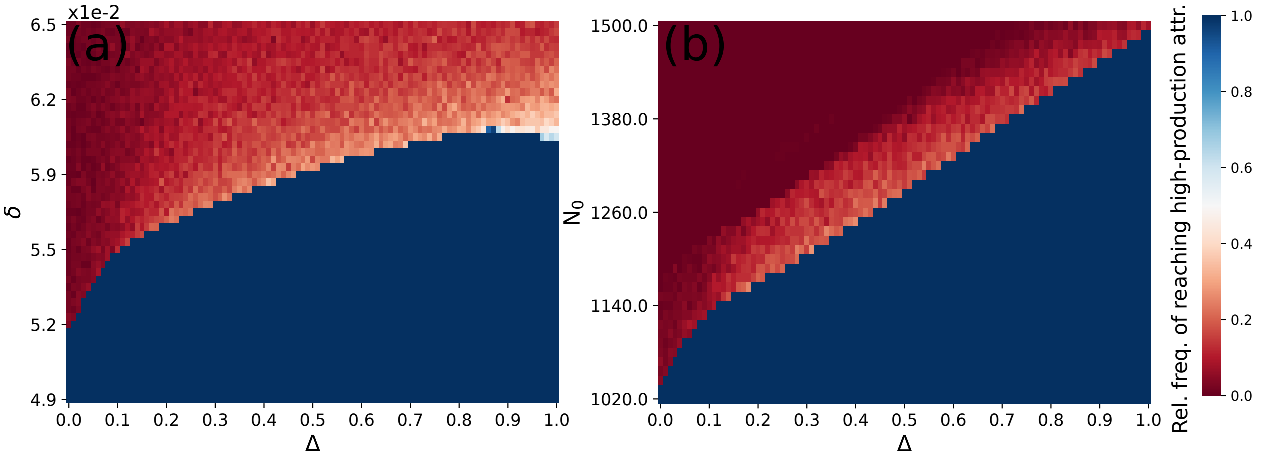

Figure A4: *(a), (b) Relative frequencies of reaching the high-production attractor, as a function of the trait difference* Δ *and the dilution rate* δ *and the nutrient inflow concentration* N_0*, respectively, for* $h = 1.1$*. Each of the points shows the relative frequency of reaching the high-production attractor, sampling* 200 *random initial conditions. The patterns described in the main text, as well as in Figure A3 are also present. Increasing* δ *further leads to extinction of one of the top species.*

A.2 Varying the body mass ratio between trophic levels

Using the standard parameter values defined in Table 1 (main text), but with a mass ratio of 500 instead of 1000, also shows a similar trend as found in Figure 5 (main text), underlining the robustness of our results (Figure A5).

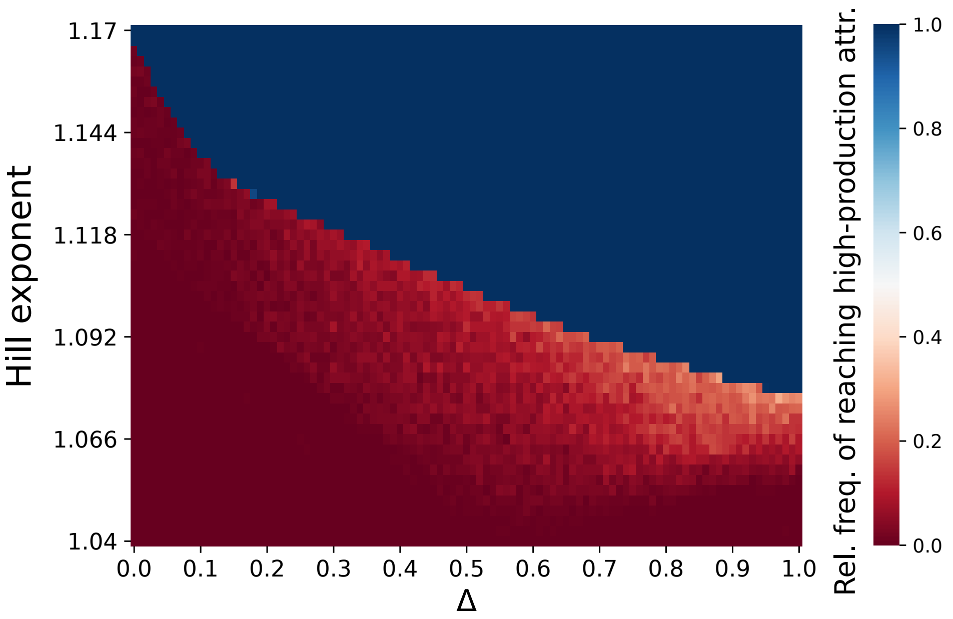

Figure A5: *Relative frequencies of reaching the high-production attractor, as a function of the trait difference* Δ *and the Hill exponents* $h = \eta$*, for a body mass ratio of* 500 *instead of* 1000 *between all trophic levels. Each of the points shows the relative frequency of reaching the high-production attractor, sampling* 200 *random initial conditions. The patterns described in the main text (Figure 5) are again present. To compensate for the higher growth rate of the top species when the body mass ratio is lowered, the dilution rate* δ *has been slightly increased to* 0.065 *instead of* 0.055*. All other parameters are the standard ones given in Table 1.*

However, lowering the body mass ratio between adjacent trophic levels further down to 100, we find that only one attractor exists over the whole range of Δ (Figures A6a & A6b). Interestingly, this single attractor changes continuously from appearing like the low-production attractor when Δ is low (Figure A6a), to resembling the high-production attractor when Δ is high (Figure A6b). This means that, as trait variation increases, the mean free nutrient level decreases, the mean biomass at the top level increases, and the temporal variability decreases. Hence, in the case where there is only one attractor, the relevant conclusion are still supported.

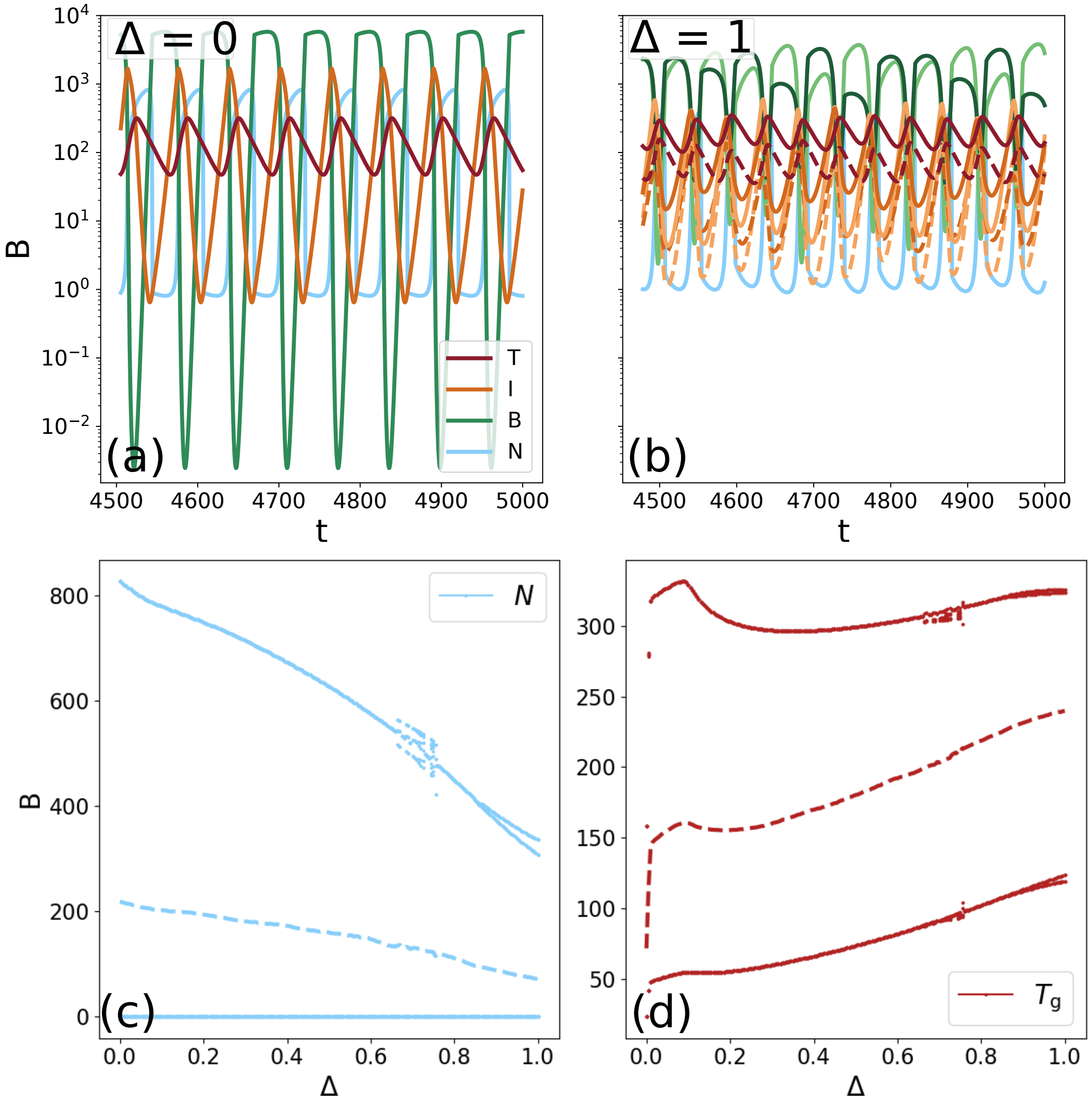

Figure A6: *(a), (b): Biomass dynamics for the linear chain ($\Delta = 0$) and fully separated web ($\Delta = 1$), respectively, when the body mass ratio is set to* 100*, for $h = 1.05$. (c), (d) Bifurcation diagram showing only one single attractor exists over the whole range of Δ, for N and T_n respectively (T_s shows a similar upward trend). The dashed line in the middle shows the mean level. As Δ is increased from* 0 *to* 1 *the mean free nutrient level drops from $\approx 220\,\mu g\,N/l$ to $\approx 70\,\mu g\,N/l$, while the mean total biomass at the top level increases from $\approx 140\,\mu g\,C/l$ to $\approx 270\,\mu g\,C/l$.*

Lastly, in order to properly compensate for the increased grazing pressure when the mass ratios are lowered in a chemostat model, we investigated a different model structure where the death rate of each species is also linked to their body mass. In this way, an increase in the maximal grazing rate of a species is accompanied by an increased death rate (Figure A7).

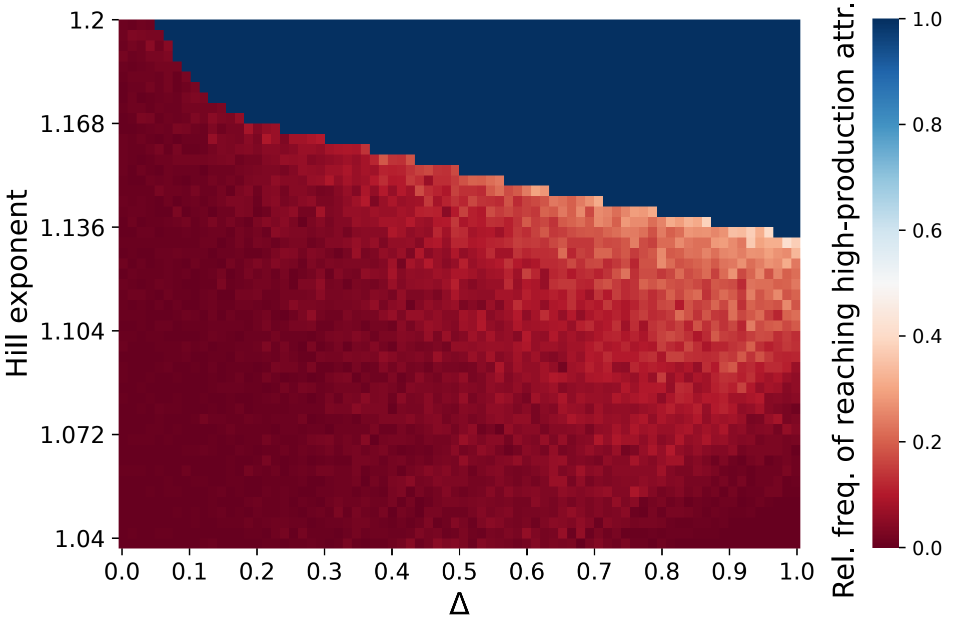

Figure A7: *Relative frequencies of reaching the high-production attractor, as a function of the trait difference Δ and the Hill exponents $h = \eta$, for a body mass ratio of* 200*, with the dilution rate δ scaled allometrically. In this case, the species on each trophic level get a distinct death rate which depends on their body mass and their growth rate. Specifically, the death rates d for the different species are defined as follows:*

$$\begin{aligned} I_1 \text{ and } I_3: &\quad d = \delta\, r_1' \,(200/1000)^{-\lambda} \\ I_2 \text{ and } I_4: &\quad d = \delta\, r_2' \,(200/1000)^{-\lambda} \\ T_1: &\quad d = \delta\, r_1' \,(200/1000)^{-2\lambda} \\ T_2: &\quad d = \delta\, r_2' \,(200/1000)^{-2\lambda}, \end{aligned}$$

where 200 *is the body mass ratio used for making the figure, and* 1000 *is the standard body mass ratio used in the study. In this way species that grow faster get a higher death rate. Each of the points shows the relative frequency of reaching the high-production attractor, sampling* 200 *random initial conditions. The patterns described in the main text (Figure 5) are again present. All other parameters are the standard ones given in Table 1.*

B Food web when $\Delta = 0$

In this appendix, the details and meaning of the transition from a food chain with 3 elements (Fig. B1) to a food web with 9 elements, where all the species on a trophic level are equal, will be presented. It is a priori not clear how these two systems are related, but it can be shown easily that the $\Delta = 0$ food web in Fig. B1b does indeed describe a food chain, but with a different half-saturation constant in the predator-prey functional response.

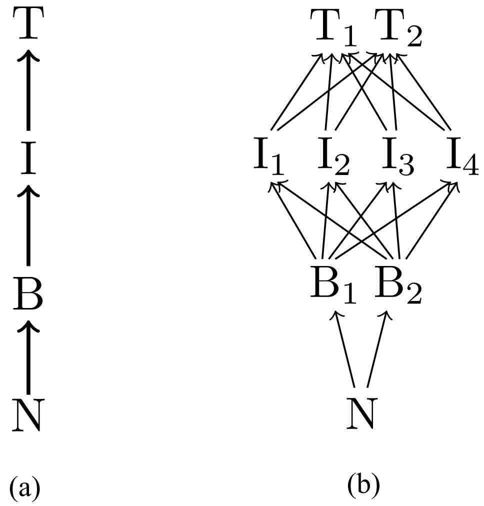

Figure B1: *(a) A simple linear tritrophic chain, described by* 4 *equations. (b) Food web where all species on a trophic level are exactly equal (*$\Delta = 0$*), described by* 9 *equations.*

As the equations are somewhat cumbersome when two species are interacting with four species and vice-versa, let us calculate what happens for a simpler toy system of two resources (R) and one consumer C:

$$\begin{cases} \dot{R}_1 &= r\,R_1 - \dfrac{R_1^h}{R_1^h + R_2^h + M^h}\,C \\ \dot{R}_2 &= r\,R_2 - \dfrac{R_2^h}{R_1^h + R_2^h + M^h}\,C \\ \dot{C} &= e\,\dfrac{R_1^h + R_2^h}{R_1^h + R_2^h + M^h}\,C - d\,C \end{cases} \tag{1}$$

where r is the resources' growth rate, h the Hill-exponent of the predator-prey interaction's functional response, M its half-saturation constant, and d the consumer's death rate. Assume now that the two resources are actually the same species, i.e., $R_1 + R_2 = R$[1], this gives:

[1]This leads to a somewhat inconsistent description, as the mechanism leading to the grazing suppression at low

$$\begin{cases} \dot{R} &= \dot{R}_1 + \dot{R}_2 = r\,R - \dfrac{R_1^h + R_2^h}{R_1^h + R_2^h + M^h}\,C \\ \dot{C} &= e\,\dfrac{R_1^h + R_2^h}{R_1^h + R_2^h + M^h}\,C - d\,C. \end{cases} \tag{2}$$

Assuming in addition that $R_1 = R_2$ (which we are free to do so when setting the initial conditions):

$$\begin{cases} \dot{R} &= r\,R - \dfrac{\left(\frac{R}{2}\right)^h + \left(\frac{R}{2}\right)^h}{\left(\frac{R}{2}\right)^h + \left(\frac{R}{2}\right)^h + M^h}\,C \\ \dot{C} &= e\,\dfrac{\left(\frac{R}{2}\right)^h + \left(\frac{R}{2}\right)^h}{\left(\frac{R}{2}\right)^h + \left(\frac{R}{2}\right)^h + M^h}\,C - d\,C. \end{cases} \tag{3}$$

Rewriting $\left(\frac{R}{2}\right)^h + \left(\frac{R}{2}\right)^h = 2^{1-h}R^h$ gives:

$$\begin{cases} \dot{R} &= r\,R - \dfrac{R^h}{R^h + \left(2^{\frac{h-1}{h}}M\right)^h}\,C \\ \dot{C} &= e\,\dfrac{R^h}{R^h + \left(2^{\frac{h-1}{h}}M\right)^h}\,C - d\,C. \end{cases} \tag{4}$$

This shows that a system with 2 identical resources and one consumer with a generalized Holling-Type-III predator-prey functional response does in fact describe a food chain, but with a different half-saturation constant: $M \to 2^{(h-1)/h}M$.

The above reasoning is easily applied to the more complex case shown in Figure B1. The 'web' of nine equations:

---

resource densities requires either the predator to be able to distinguish the different resource types, or the prey to have a particular means of more efficiently escaping predation at low resource densities. Both options are not possible when the two resources are the same species, since it implies they cannot be distinguished in any way, and would e.g. use similar hiding spaces to escape predation.

$$\begin{cases} \dot{N} &= \delta\,(N - N_0) - \dfrac{c_N}{c_C}\, r\, \sum_i B_i \\ \dot{B}_i &= r\, B_i - g_i \sum_j I_j - \delta B_i \\ \dot{I}_j &= e \sum_i g_i I_j - \gamma_j \sum_i T_i - \delta\, I_j \\ \dot{T}_i &= \epsilon \sum_j \gamma_j T_i - \delta\, T_i \end{cases} \tag{5}$$

with $i \in \{1, 2\}$, $j \in \{1, 2, 3, 4\}$, and

$$r = r' \frac{N}{N + h_N} \tag{6}$$

$$g_i = g' \frac{B_i^h}{\sum_{i'} B_{i'}^h + M^h} \tag{7}$$

$$\gamma_j = \gamma' \frac{I_j^\eta}{\sum_{j'} I_{j'}^\eta + \mu^\eta} \tag{8}$$

can easily be calculated to correspond to a linear chain described by four equations:

$$\begin{cases} \dot{N} &= \delta\,(N_0 - N) - \dfrac{c_N}{c_C} \cdot r\, B \\ \dot{B} &= rB - g\, I - \delta\, B \\ \dot{I} &= e \cdot g\, I - \gamma\, T - \delta\, I \\ \dot{T} &= \epsilon \cdot \gamma\, T - \delta\, T, \end{cases} \tag{9}$$

with

$$r = r' \frac{N}{N + h_N} \tag{10}$$

$$g = g' \frac{B^h}{B^h + M^h} \tag{11}$$

$$\gamma = \gamma' \frac{I^\eta}{I^\eta + \mu^\eta}, \tag{12}$$

when

$$B_1 = B_2, \qquad I_1 = I_2 = I_3 = I_4, \qquad T_1 = T_2, \tag{13}$$

by setting

$$\sum_i B_i = B, \qquad \sum_j I_j = I, \qquad \sum_i T_i = T, \tag{14}$$

and

$$M \to 2^{(h-1)/h} M, \qquad \mu \to 4^{(h-1)/h} \mu. \tag{15}$$

Values of h used in our study are very close to one, hence, the difference is not very large. For example, $h = 1.1$ implies roughly $M \to 1.07\, M$ and $\mu \to 1.13\, \mu$.

C Additional information

This appendix contains some extra figures and tables supporting the results discussed in the main text. The bifurcation diagrams of all other species as well as the nutrients (Figures C1 & C2) confirm that the information extracted from the one shown in the main text are universal. The frequency spectra from which the relevant timescales were identified are shown in Figure C3. Figure C4 shows the long chaotic transient which is present right after the boundary crisis of the high-production attractor.

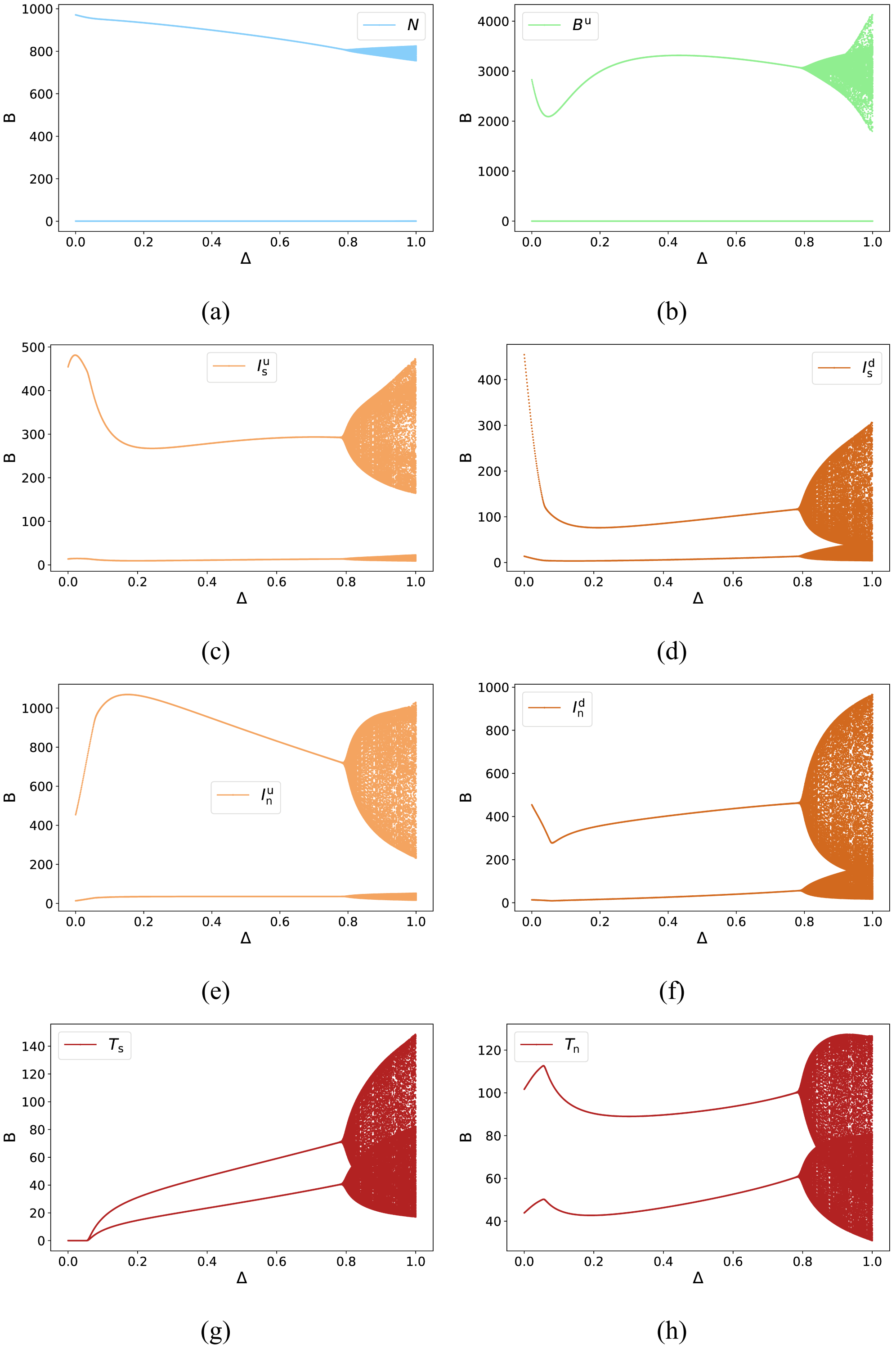

Figure C1: *Bifurcation diagrams of the low-production attractor for $h = \eta = 1.05$, as the trait difference parameter Δ is varied from 0 to 1. For small to intermediate sizes of the maximum trait difference, the oscillations are simple in the sense that the maxima and minima are always at the same height. At $\Delta \approx 0.8$ a transition occurs to a region where the oscillations become complex, as indicated by the varying locations of the maxima and minima.*

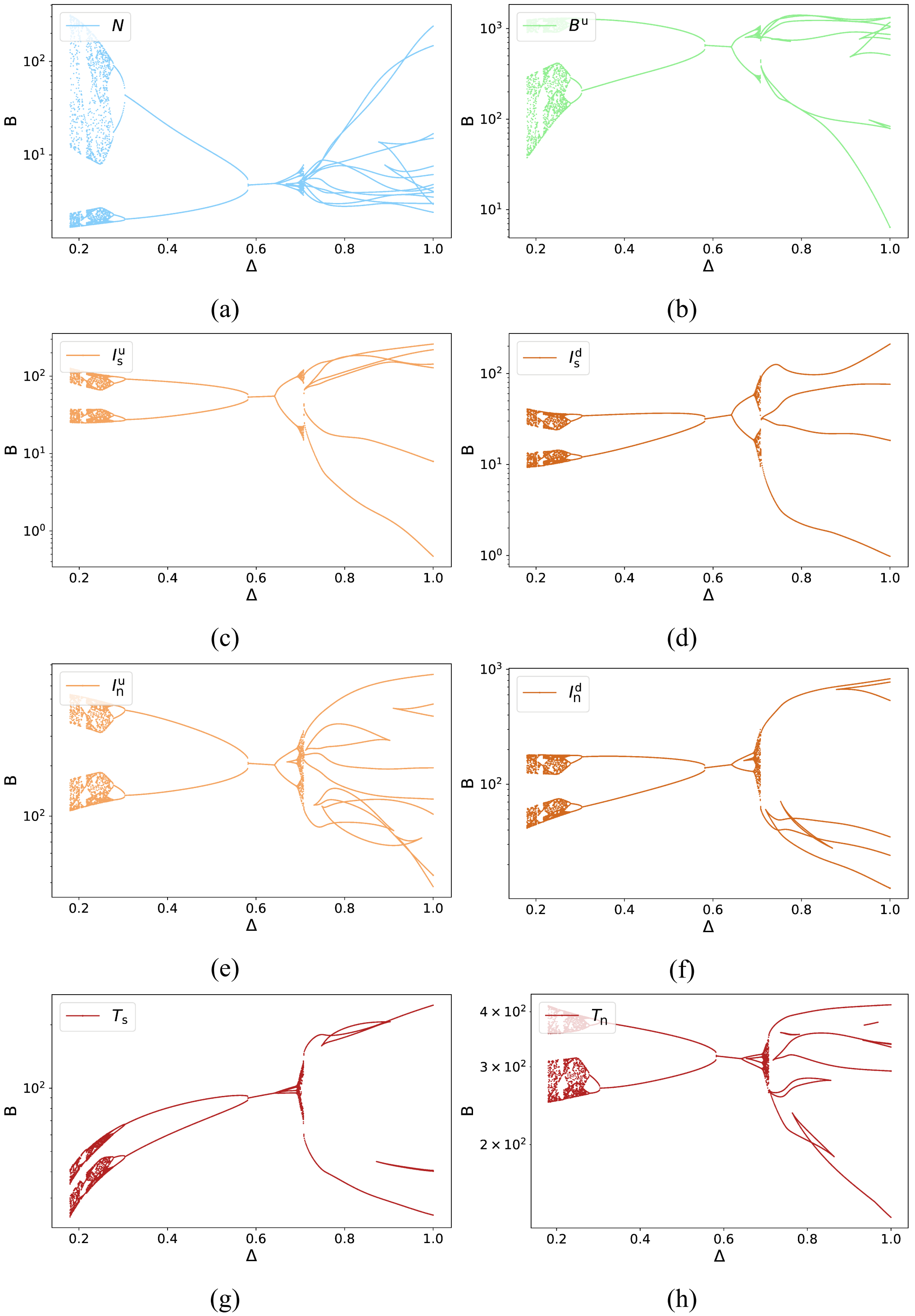

Figure C2: *Bifurcation diagrams of the high-production attractor for* $h = \eta = 1.05$*, as the trait difference parameter* Δ *is varied from* 0 *to* 1*. The attractor disappears for* $\Delta \lessapprox 0.18$*, and undergoes several bifurcations in the region* $0.18 \lessapprox \Delta \leq 1$.

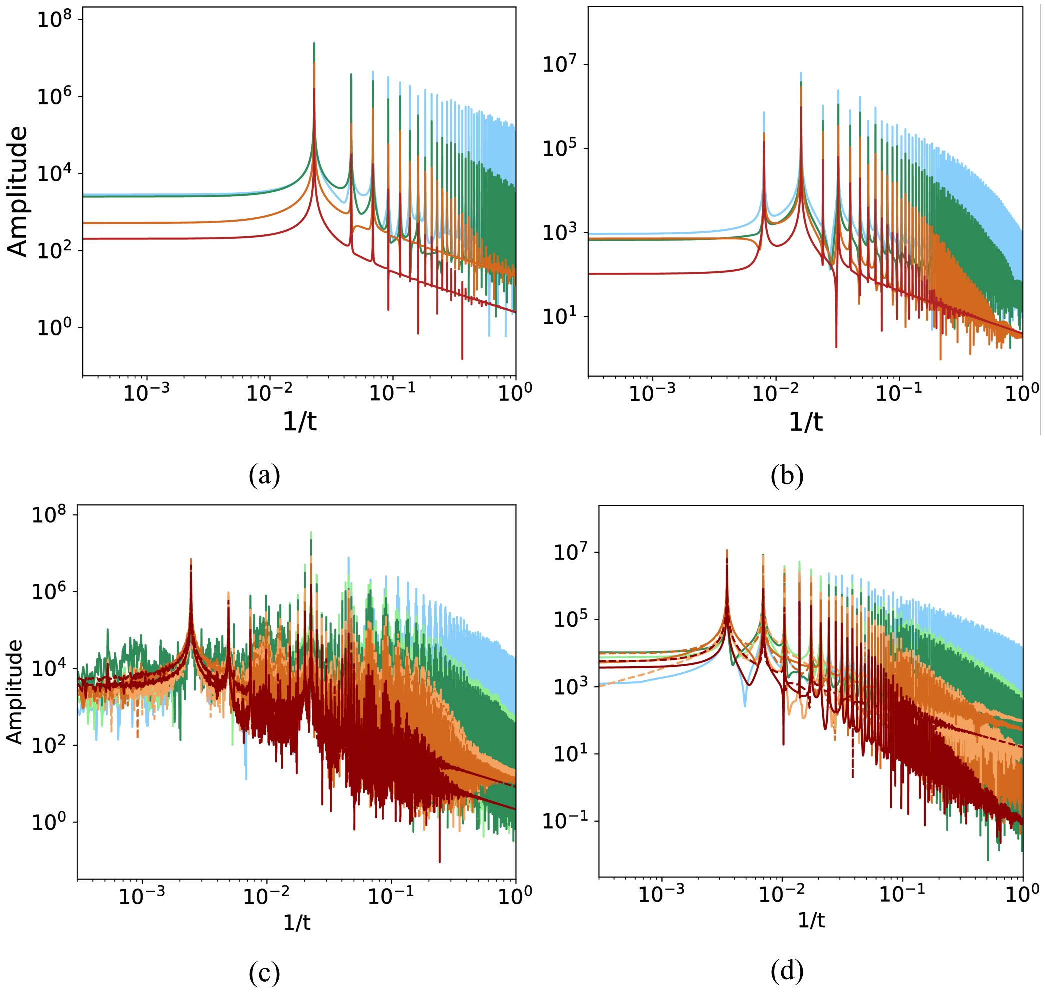

Figure C3: *Fourier spectra of the linear chain ($\Delta = 0$), for $h = \eta = 1.1$ (top), and for the separated food web ($\Delta = 1$), for $h = \eta = 1.05$ (bottom). The left-most figures show the low-production attractor, and the rightmost the high-production attractor. These spectra show that the trait dynamics impose a much longer timescale than the one already present in the linear chain.*

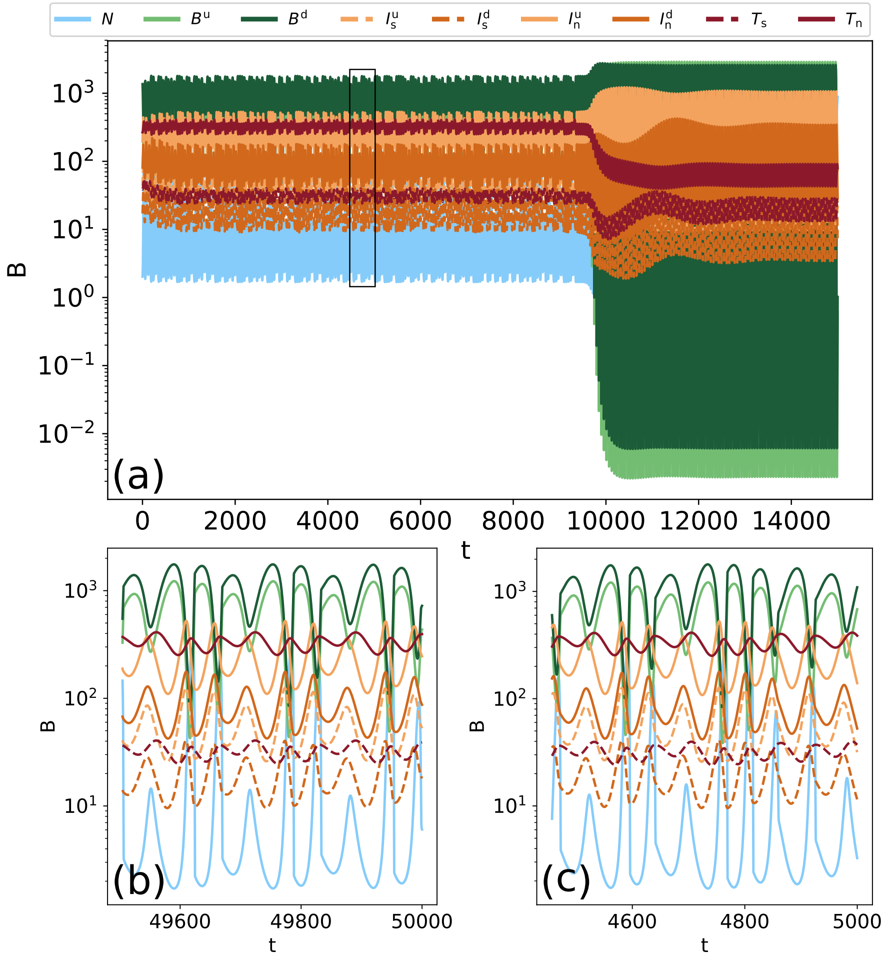

Figure C4: *(a): Biomass dynamics for* $h = \eta = 1.05$ *and* $\Delta = 0.177$*, slightly smaller than the value where the high-production attractor disappears. A long chaotic transient is observed before the system suddenly accelerates towards the only remaining attractor, the low-production attractor. Figure (b) shows the dynamics for* $\Delta = 0.18$ *where the attractor still exists, and demonstrates that they are virtually indistinguishable from those on the transient shown in Figure (c) (close-up of the box in Figure (a)).*

	$\Delta = 0$		$\Delta = 1$	
	HP	LP	HP	LP
$(P/B)_{\text{Top}}$	5.5	5.5	5.6	5.5
$(P/B)_{\text{Basal}}$	21.8	16.8	30.3	20.2
$P_{\text{Top}}/P_{\text{Basal}}$	6.0	3.9	6.3	3.1

Table C1: *Production (P) to Biomass (B) ratios of the Top and Basal trophic levels, for the linear chain ($\Delta = 0$) and the maximally separated food web (Δ= 1), for both the high-production (HP) and the low-production (LP) states. The bottom row shows that the top-production per unit of basal production also increases on the high-production state, resulting in a more efficient system.*

References

Brose, U., T. Jonsson, E. L. Berlow, P. Warren, C. Banasek-Richter, L. F. Bersier, J. L. Blanchard, T. Brey, S. R. Carpenter, M. F. C. Blandenier, L. Cushing, H. A. Dawah, T. Dell, F. Edwards, S. Harper-Smith, U. Jacob, M. E. Ledger, N. D. Martinez, J. Memmott, K. Mintenbeck, J. K. Pinnegar, B. C. Rall, T. S. Rayner, D. C. Reuman, L. Ruess, W. Ulrich, R. J. Williams, G. Woodward, and J. E. Cohen. 2006. Consumer-resource body-size relationships in natural food webs. Ecology 87:2411–2417. doi:10.1890/0012-9658(2006)87[2411:CBRINF]2.0.CO;2.
